# Resilience in the global food system

**DOI:** 10.1088/1748-9326/aa5730

**Published:** 2017-02-09

**Authors:** David A. Seekell, Joel Carr, Jampel Dell’Angelo, Paolo D’Odorico, Marianela Fader, Jessica A. Gephart, Matti Kummu, Nicholas Magliocca, Miina Porkka, Michael J. Puma, Zak Ratajczak, Maria Cristina Rulli, Samir Suweis, Alessandro Tavoni

**Affiliations:** 1) Department of Ecology and Environmental Science, Umeå University, Umeå, Sweden; 2) Department of Environmental Sciences, University of Virginia, Charlottesville, Virginia; 3) National Center for Socio-Environmental Synthesis, University of Maryland, Annapolis, Maryland; 4) International Centre for Water Resources and Global Change (UNESCO), hosted by the Federal Institute of Hydrology, Koblenz, Germany; 5) Water & Development Research Group, Aalto University, Aalto, Finland; 6) Center for Climate Systems Research & Center for Climate and Life, Columbia University, NASA Goddard Institute for Space Studies, New York, New York; 7) Dipartimento di Ingegneria Civile e Ambientale, Politecnico di Milano, Milano, Italy; 8) Department of Physics and Astronomy, University of Padova, Padova, Italy; 9) Grantham Institute on Climate Change and the Environment, London School of Economics, London, United Kingdom

**Keywords:** food security, resilience, food systems, food production, sustainability

## Abstract

Ensuring food security requires food production and distribution systems function throughout disruptions. Understanding the factors that contribute to the global food system’s ability to respond and adapt to such disruptions (i.e. resilience) is critical for understanding the long-term sustainability of human populations. Variable impacts of production shocks on food supply between countries indicate a need for national-scale resilience indicators that can provide global comparisons. However, methods for tracking changes in resilience have had limited application to food systems. We developed an indicator-based analysis of food systems resilience for the years 1992–2011. Our approach is based on three dimensions of resilience: socio-economic access to food in terms of income of the poorest quintile relative to food prices, biophysical capacity to intensify or extensify food production, and the magnitude and diversity of current domestic food production. The socio-economic indicator has large variability, but with low values concentrated in Africa and Asia. The biophysical capacity indicator is highest in Africa and Eastern Europe, in part because of high potential for extensification of cropland and for yield gap closure in cultivated areas. However, the biophysical capacity indicator has declined globally in recent years. The production diversity indicator has increased slightly, with a relatively even geographic distribution. Few countries had exclusively high or low values for all indicators. Collectively, these results are the basis for global comparisons of resilience between nations, and provide necessary context for developing generalizations about the resilience in the global food system.

## 1 Introduction

Achieving food security is central to the United Nations (UN) Sustainable Development Goals. The UN Food and Agriculture Organization (FAO) defines food security as “a situation that exists when all people, at all times, have physical, social and economic access to sufficient, safe and nutritious food that meets their dietary needs and food preferences for an active and healthy life” (FAO 2001). As a result, ensuring food security requires that food production and distribution systems function despite potential disruptions. It also requires that all people have economic access to a sufficient amount of food to satisfy their nutritional needs. Meeting this goal in the face of a growing human population, shifting diets, limited natural resources, climate change, and environmental variability is a major challenge of our time (Godfray *et al* 2010; Foley *et al* 2011).

The ability of a food system to respond and adapt to disruptions, while maintaining its function, describes the system’s resilience (Pingali *et al* 2005, Schipanski *et al* 2015). Like all complex social-ecological systems, resilience within food systems cannot be evaluated at a single scale (Folke *et al* 2010, Béné *et al* 2016). Consequently, local, global, and cross-scale interactions must be included when evaluating resilience within the increasingly globalized food system (Porkka *et al* 2013, D’Odorico *et al* 2014, Gephart and Pace 2015, MacDonald *et al* 2015). Further, food systems must be evaluated with respect to both the short-term responses and the longer-term factors that contribute to resilience (Pingali *et al* 2005; Béné *et al* 2016).

At the local scale, research on food systems resilience has mostly focused on disaster response case studies and detailed evaluations of infrastructure, governance, and social networks (Béné *et al* 2016). These analyses help identify features of resilient systems including specific mechanisms that allow them to respond and adapt to disruptions. For example, in 1992–1993 food production in southern Africa was adversely impacted by a drought related to El Niño, but there was no regional food crisis. In 2002–2003 a similar drought caused a regional famine, and this contrast has been interpreted as indicative of declining resilience related to conflicts and adverse impacts of the HIV/AIDS pandemic on social and government institutions (Pingali *et al* 2005).

At the global level, resilience research has a different focus, evaluating economic patterns and relationships rather than food security for individuals or households. Global-scale resilience has been studied by tracking how shocks to food system propagate internationally (Marchand *et al* 2016). For instance, extreme environmental conditions in 2007 and 2010 caused agricultural failures in some countries. Export bans meant to protect populations in producing countries came at the expense of nations reliant on trade to balance their food needs (Fader *et al* 2013, Baldos and Hertel 2015). Food prices rose sharply, increasing the numbers of undernourished people and creating social unrest including food riots (Fader *et al* 2013, Lagi *et al* 2011, Berazneva and Lee 2013, Baldos and Hertel 2015). Studies combining population dynamics, food production, and trade have found that the global food system has become increasingly fragile (Fraser *et al* 2005, D’Odorico *et al* 2010, Suweis *et al* 2015, Puma *et al* 2015, Marchand *et al* 2016). Global-scale factors like trade may enhance food security locally but reduce the resilience of the global food system, while local scale factors that include more proximal drivers of food security - such as grain reserves or the potential to increase local food production - act within the context of global scale patterns and processes (Fraser *et al* 2005, D’Odorico *et al* 2010, Baum *et al* 2015, Puma *et al* 2015, Fader *et al* 2016, Gephart *et al* 2016, Marchand *et al* 2016, Gephart *et al* 2017).

In order to track the evolution and current state of resilience within the global food system, we collected national level indicators at multiple time points to evaluate the overall state and trajectory of three dimensions of country-level resilience. The indicators characterize: socio-economic access to food in terms of income of the poorest quintile relative to average food prices, biophysical capacity to sustainably intensify or extensify food production, and magnitude and diversity of domestic food production. Here, we describe the geographic and temporal (1992–2011) patterns of these resilience indicators, and evaluate the indicators for potential redundancies. Our analysis provides an opportunity for global-scale generalizations and comparisons of resilience at the country level, and the context necessary for developing cross-scale analyses of food systems resilience.

## 2. Methods

### 2.1 Conceptual Basis

The resilience concept was popularized through studies of ecosystems with alternative states. In this context, resilience describes an ecosystem’s ability to remain in a particular state under perturbations (Holling 1973, Folke *et al* 2010). Since its introduction in ecology, resilience theory has been applied to a wide range of complex systems and has adopted a more general definition of “the capacity of a system to absorb disturbance and reorganize while undergoing change so as to still retain essentially the same function, structure, identity, and feedbacks” (Walker *et al* 2004). Operationally, the concept has been used in several ways, including as a metaphor associated with sustainability, a feature of dynamic models, and a quantifiable field measurement (Carpenter *et al* 2001).

The resilience concept can be applied across multiple scales (Béné *et al* 2016). For example, factors influencing household-level resilience include the maintenance or sale of assets like livestock and dietary variation of meals (Misselhorn 2005). At the national scale, food security is influenced by factors like margins of self-sufficiency and financial ability to balance food deficits with imports from other countries (e.g. Suweis *et al* 2015). Other attributes including production diversity and the size of national grain reserves contribute to the ability to avoid or cope with disruptions and are therefore used as general indicators of resilience (e.g. Walker and Salt 2006). Finally, at the global level, factors including the structure of trade networks influence the propagation of perturbations between countries and overall stability or fragility of the globalized food system (e.g. D’Odorico *et al* 2010, Puma *et al* 2015, Gephart *et al* 2016, Marchand *et al* 2016). These factors can change at short or long time scales (Béné *et al* 2016).

Quantitative methods for tracking changes in resilience remain best developed in ecology (e.g. van Nes and Scheffer 2007, Scheffer *et al* 2009, Carpenter *et al* 2011). Key ecosystem variables are monitored and individually evaluated for reductions in the rate of return to equilibrium after perturbations – known as critical slowing down – measured as changes in autocorrelation and variance. These methods are effective at evaluating resilience in a diverse array of ecosystems (Drake *et al* 2010, Carpenter *et al* 2011, Dakos *et al* 2012, Kéfi *et al* 2014). These metrics have subsequently been extended to track changes in the resilience of socio-ecological networks (Suweis and D’Odorico 2014). The global food system can be conceptualized as a complex network where countries are nodes with endogenously resilient food production systems and consumption, where international trade connects nodes and acts as another source of resilience. The network theory framework has allowed critical slowing down and related approaches to evaluating changes in resilience to be applied to the global food system (e.g. D’Odorico *et al* 2010, Suweis *et al* 2015). However, there are important limitations to applying the resilience metrics developed by ecologists to food systems. Specifically, application of critical slowing down based resilience metrics tested by ecologists assumes there is no difference in key functional structure between social institutions and ecosystem processes, an assumption that is contested by some social scientists (Adger 2000, Barrett and Constas 2014, Olsson *et al* 2015, Béné *et al* 2016). Additionally, critical slowing down based resilience metrics only indicate that change may occur; they do not discriminate between impending shifts to conditions of decreased human wellbeing versus transitions to improved human well being (Bauch *et al* 2016). Hence, existing approaches cannot yet fully describe patterns and processes relative to resilience in the global food system (Béné *et al* 2016).

A pragmatic way to complement critical slowing down based resilience metrics is to develop an index-based analysis of the capacity of countries to handle shocks (e.g. Allison *et al* 2009, Fader *et al* 2016, Marchand *et al* 2016). Index based methods rely on surrogate measures that reflect aspects of resilience that are difficult to measure or model (Adger 2000, Carpenter *et al* 2005). Additionally, directional change in indicators can have explicit interpretations, whereas critical slowing down based methods are more ambiguous about the nature of change (Bauch *et al* 2016). Here, we focus on developing indicators for national-scale resilience. We have selected the national scale for four reasons:

1. Domestic and foreign policies are set at the national level and thus provide the context in which proximal causes and consequences of individual food security or lack thereof occur.
2. A recent review found that most analyses of resilience in food systems are at the household or community scale and broader scale analyses are lacking (Béné *et al* 2016).
3. National scale indicators of food security are available with global coverage. Finer scale (e.g. household) metrics are available, but typically not with global coverage (Naiken 2003).
4. Many indicators of food security at the national scale are available as time series, allowing us to track inter-annual variability and longer-term changes in ways not possible at smaller scales.

**Figure 1.**
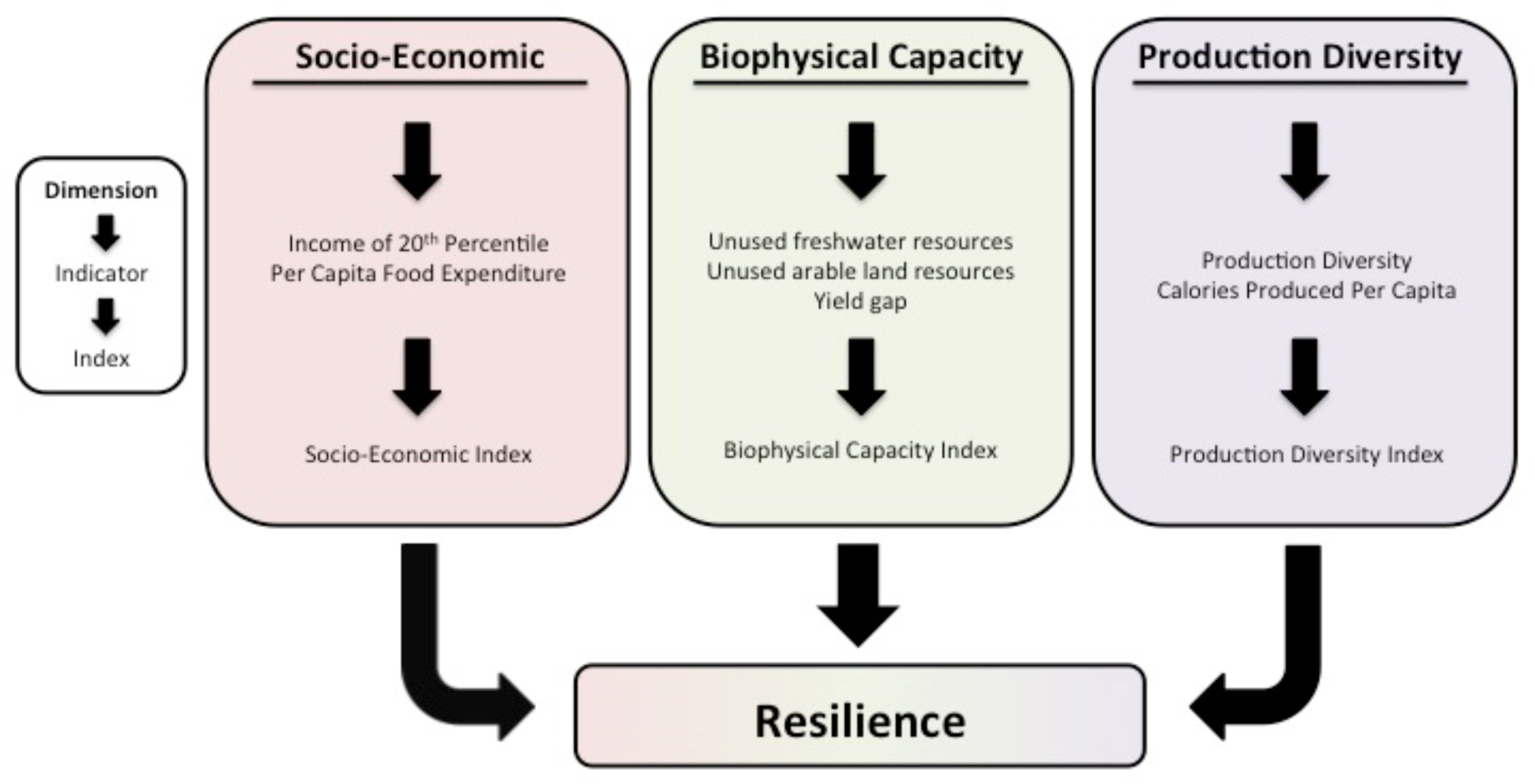
Three dimensions of resilience considered in this analysis. A national-scale index was created to track each dimension. Each index has global coverage. These dimensions reflect the FAO definition of food security, specifically that all people have physical (biophysical capacity), social and economic access (socio-economic index) to sufficient, safe and nutritious food that meets their dietary needs and food preferences for an active and healthy life (production diversity index).

We consider three main dimensions of resilience within an index framework: the ability to access food which is based on social and economic factors, biophysical capacity to increase food production through sustainable intensification or extensification, and the magnitude and diversity of domestic food production (Figure 1). For each dimension, we created an aggregate index of resilience based on two to three key indicators. We described these indicators and indices in detail below and have made the indicators available on Github (doi: 10.5281/zenodo.192394).

### 2.2 Access to Food

Access to food is chiefly a socio-economic issue related to prices and income (Barrett 2010). Typically, a country’s poor are most likely to suffer from food insecurity (Bohle *et al* 1994, Timmer 2000). Being poor does not necessarily imply food insecurity, but it does limit options during periods of price spikes, crop failures for subsistence farmers, or loss of assets such as livestock (Timmer 2000). Therefore, we consider resilience to be higher in countries where the poor have higher income relative to food prices, compared to countries where the poor have low incomes relative to food prices (Timmer 2000). Other socio-economic factors including levels of education, especially for women, and investments in infrastructure influence food security and resilience at local scales, but we focus on income related factors here because these are thought to be the primary influence on food security when evaluated at broad scales (Timmer 2000, Godfray *et al* 2010).

We calculated an index of socio-economic access to food based on two indicators: the average income of the lowest 20% of each country’s income distribution (per capita) and average per capita food expenditure (cf. Timmer 2000). This metric reflects a measure of liquid assets that can be readily exchanged for food. Estimates of the income of the lowest 20% of the population are based on several sources. Most values were based on income data from the World Bank, estimated using their PovcalNet tool (http://iresearch.worldbank.org/PovcalNet/index.htm). In some cases, there were not enough values in the World Bank dataset, so we used data from the United Nations University WIID 3.3 database (https://www.wider.unu.edu/download/WIID3.3). Average food expenditure per capita was based on the FAO Domestic Food Price Level Index. This index represents the price of food in each country relative to the United States in purchasing power parity terms. Data were not available for all years, so we used logarithmic interpolation to complete time series. For 70 countries, this interpolation was based on five observations during the period 1992–2014. For 24 countries it was based on four observations, but with at least one observation before 1990. We combined the income and food price metrics into a single index by taking the ratio of income to food price. Lower values suggest increasing trade-offs with other critical expenditures (e.g., housing) and reduced ability to make-up caloric deficits through food purchases.

### 2.3 Biophysical Capacity to Produce Food

We conceptualize the biophysical capacity to produce food as a function of area of suitable, uncultivated land, untapped freshwater resources, and potential for closure in agricultural yield gaps (percentage of actual production divided by potential production). Increasing either of these factors will increase the biophysical capacity of countries to ramp-up food production through extensification (putting unused land and water resources into production) or intensification (decreasing yield gap through nutrient supply, irrigation, or utilizing new technology) in the case of increased demand or decreased production capacity (Fader *et al* 2016). Having little unused land or water resources, or no possibility to reduce yield gap, indicates limited ability to increase food production domestically. In this sense biophysical capacity contributes to resilience as a form of redundancy (e.g. Walker and Salt 2006). Intensification or extensification of agricultural production mainly occurs over longer time spans because of the time necessary to obtain capital, develop these new resources, and distribute technologies to improve yield gaps (Godfray *et al* 2010).

Here, we use a biophysical capacity index developed and described by Fader *et al* (2016). This index is based on three indicators: volume of renewable freshwater resources, availability of farmable land for agricultural extensification, and ability to intensify agriculture as indicated by yield gap (Fader *et al* 2016). Briefly, volume of freshwater resources was estimated based on data from the FAO AQUASTAT database. Unused resources were calculated as the total renewable freshwater resources minus water withdraws, environmental flow requirements, and the amount of water that is unavailable due to seasonal variability, rainfall intensity, spatial access, or lack of infrastructure. Unused arable land resources were estimated based on the HYDE 3.2 land use database (http://themasites.pbl.nl/tridion/en/themasites/hyde/) and the FAO Global Agro-Ecological Zones database. Unused arable land was calculated as total land area minus land area already used for agriculture (excluding pastures), land not suitable for agriculture, and land used for urban areas and other types of human settlement. Finally, yield gap was estimated as the difference of actual yields for a given year and the maximum yields in similar areas given ideal fertilization and irrigation minus actual production, multiplied by the spare and used areas. These maximum values were estimated following the approaches of Mueller *et al* (2012). For each factor, we compiled values for the years 1992–2011. Fader *et al* (2016) considered a variety of scenarios representing different levels of availability for unused land and water resources. For the present analysis, we consider values from the middle scenario. The values for each index were combined into an aggregate biophysical capacity measure by assuming that land and water were non-substitutable, but that yield gap was substitutable with these factors. In other words, increasing amount of available farmland does not increase biophysical capacity to produce food if there is not also available water. However, extensifying or potential for intensifying (yield gap closure) can both (or either) be used to increase biophysical capacity. This index is scaled between 0 and 1, with values less than 0.5 indicating limited water, land, or productivity redundancy and an inability to produce at least 3000 kcal d^−1^ per capita, a widely used value of dietary energy (Fader *et al* 2016).

### 2.4 Production Diversity

We consider production diversity to be related to the ability of countries to reliably meet food demand through domestic production (Pingali *et al* 2005). This means maintaining a high level of production despite (mostly) stochastic factors, such as weather variations including heat waves and drought, biotic influences including invasive species and pests, plus the consequences of local management decisions that include salinization and lost production due to over-grazing (Walker and Salt 2006, D’Odorico *et al* 2010). Average production (kcal per capita) reflects the ability of countries to meet caloric needs in a typical year, but not the resilience of countries to short-term shocks that could decrease food availability over months or years. For example, a country could have high production per capita, but if the majority of calories are from just a few commodities, then this supply stream could be vulnerable to crop-specific pests or weather outside the dominant crops’ optimum range. In general, more diverse biological systems are thought to exhibit higher aggregate stability due to species asynchrony, portfolio effects, and a number of other mechanisms (Chapin *et al* 2000, Schindler *et al* 2010, Tilman *et al* 2014). Hence, we consider countries with high production for a greater variety of crops to be more resilient than countries with low production or low diversity in production.

We calculated the “h-index” from bibliometric analyses as an index that balances indicators of total production and breadth of production (Hirsch 2005). First, we calculated the annual domestic production per capita of each commodity, *C*_*i*_, in each country:

$$C_{i}=K_{i}/P_{i}$$

where *K*_i_ is the total kcal produced by a commodity in a given year and country, and *P*_i_ is the population. *K*_i_ was determined using the FAO commodities production database (given in units of weight) and using the FAO conversion factors to express *K*_i_ in kcal (D’Odorico *et al* 2014; http://faostat.fao.org). We focus on calories instead of other nutritional characteristics (e.g. protein or micronutrient content) because it is easily comparable across countries and is also the basis for the biophysical capacity indicator (Fader et al. 2016). For the diversity analysis, we only considered primary food products, which prevents double counting of caloric production through the production of secondary products, like flours or processed animal products (D’Odorico *et al* 2014). We then calculated each country’s h-index for the years 1992–2011. All *C*_i_ were ordered from greatest to least and given a rank depending on their order in this sequence (i.e. the highest *C*_i_ has a rank 1, the second highest has a rank 2, and so on). Then, we calculated the h-index as the largest rank for which the rank is equal or less than the corresponding *C*_i_. In other words, an h-index of 20 would indicate that a country has 20 commodities that produce at least 20 kcal per capita, but not 21 commodities producing at least 21 kcal per capita. A country can only score a high h-index value if it has a production stream that has high production per capita and is also diverse. For example, a country that produced 1500 kcal per capita of corn, but then only 10 kcal per capita of nine other commodities would have an h-index of 10.

### 2.5 Evaluation of Redundancy Between Indicators

**Figure 2.**
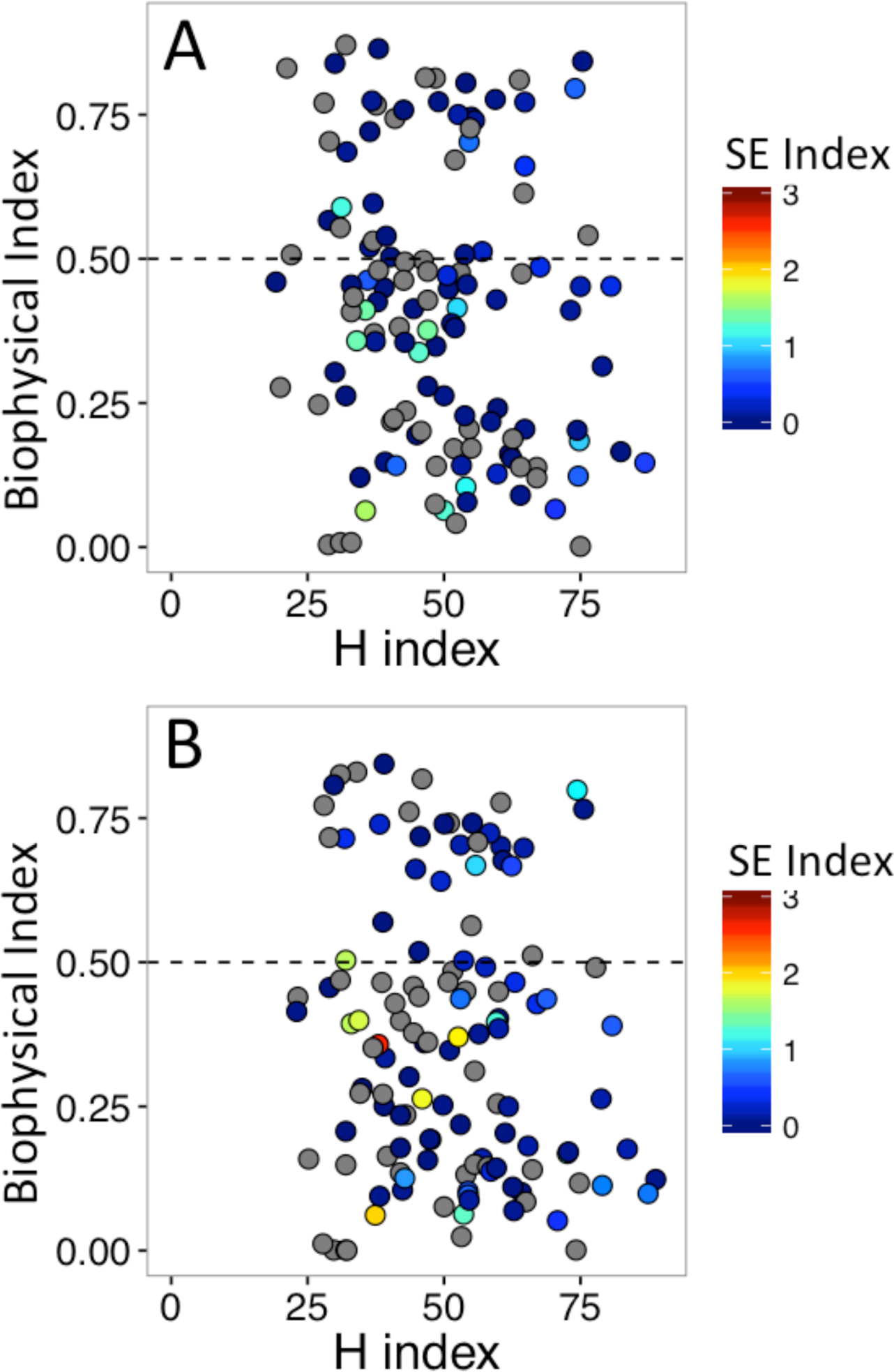
Bi-plots displaying the relationships between the biophysical capacity indicator, the production diversity indicator (h-index), and the socio-economic indicator (color bar). The dashed line represents the food security threshold for the biophysical capacity described in the main text. The upper panel displays data averaged over the period 1992–1996 and the lower panel displays data average over the period 2007–2011. Grey circles are countries where data were not available for the social economic indicator.

We evaluated the potential for redundancy between indicators using Kendall’s τ, a rank-based correlation coefficient (Kendall and Gibbons 1990). There was, at most, a minor relationship between the indicators (Figure 2). Correlations between indicators were similar for five-year averages at the beginning (1992–1996) and end (2007–2011) of the records (Table 1). In both cases there was no significant relationship between the socioeconomic and biophysical capacity indicators and no significant relationship between the biophysical capacity and production diversity index. The correlation between the socio-economic indicator and production diversity was statistically significant, but the effect size was weak at both the beginning and end of the record. This analysis indicates that these three indicators have minimal redundancy in capturing aspects of resilience.

## 3 Empirical Results: Geographic and Temporal Patterns of Resilience Indicators

**Figure 3.**
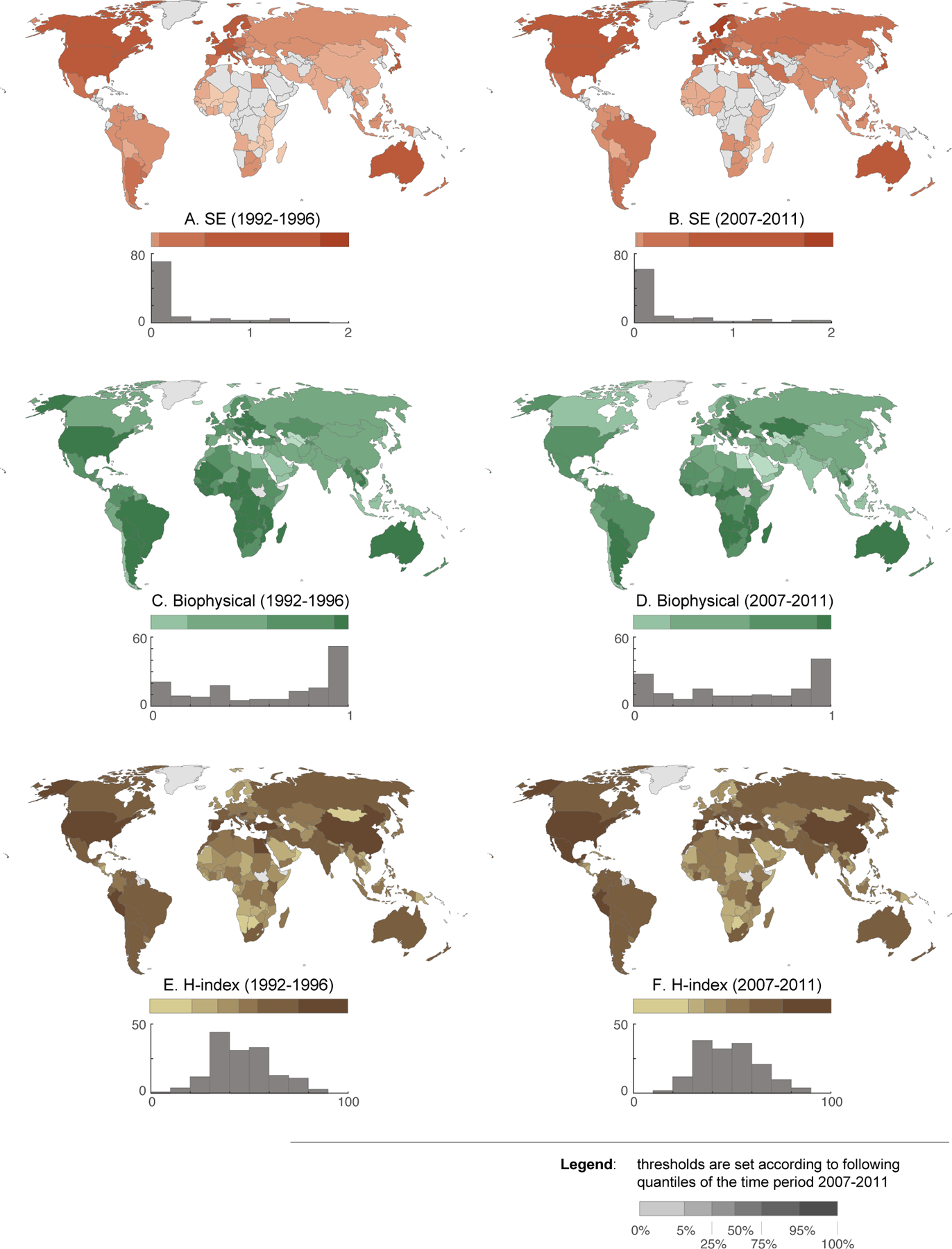
Maps of the indicators for three dimensions of resilience at the beginning (left) and end (right) of the record. Color ramps are defined based on the histogram for each panel.

**Figure 4.**
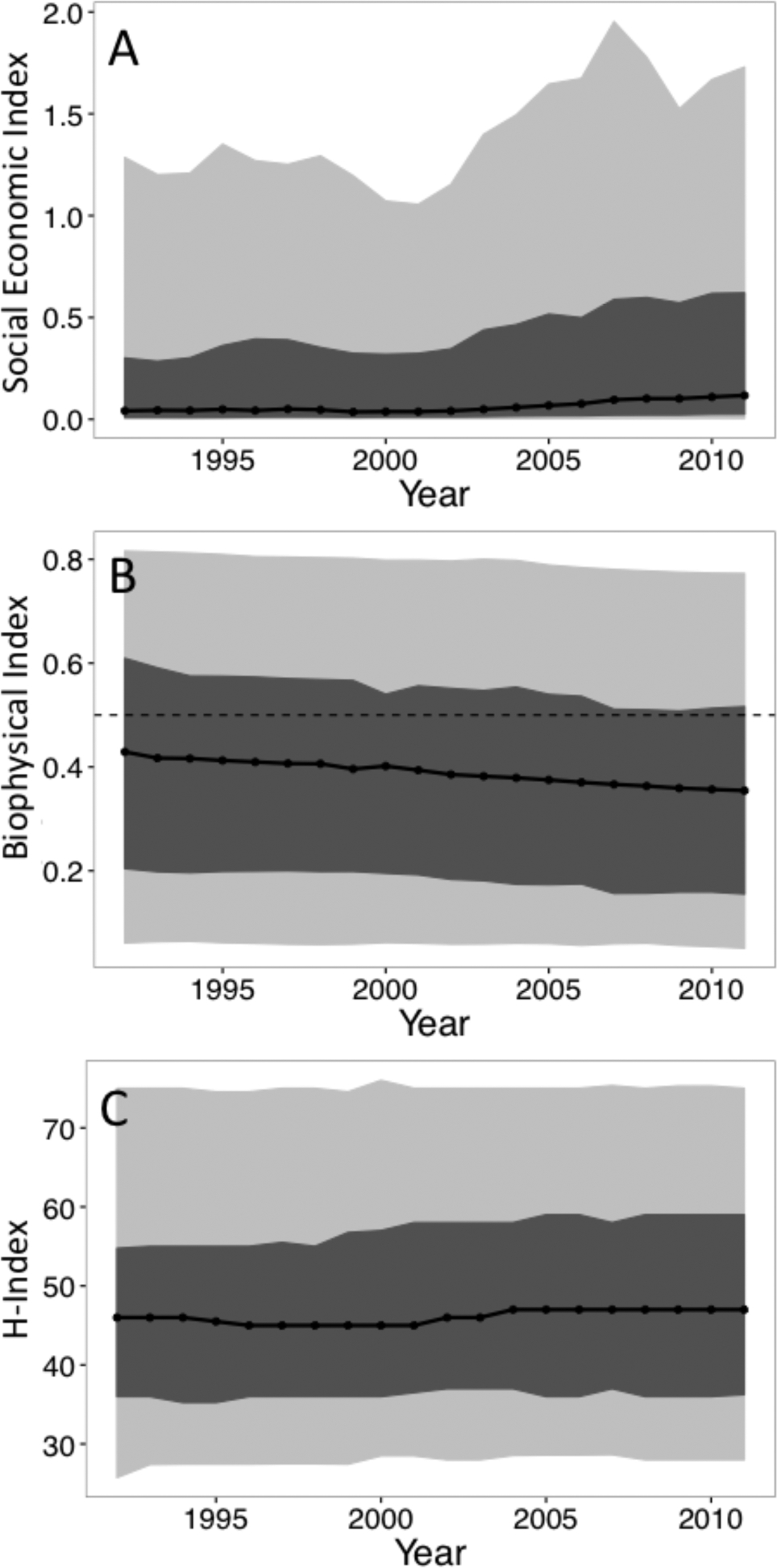
Median (black line) for the (A) socio-economic, (B) biophysical capacity, and (C) production diversity (h-index) indices. The dark gray bands are the 25th and 75th percentiles. For the socio-economic indicator, the light gray bands are the 10th and 90th percentiles. The dashed lines in panel B is a threshold value for food security describe in the main text..

We evaluated patterns and changes in the resilience indicators based on 5-year averages at the beginning (1992–1996) and end (2007–2011) of the record (Figure 3). The distribution of the socio-economic indicator was strongly right skewed throughout the record (Figure 3). Specifically, at the beginning of the record 90% of countries had socio-economic indicator values < 1, indicating that their poor earn substantially less than average food expenditures are within the country. In fact the median socio-economic indicator values was just 0.04 (Figure 4). At the end of the record, 86% of countries had socio-economic indicator values < 1 and the median indicator value had increased to 0.08 (Figure 4). Across the record, high indicator values were clustered in Western Europe and the lowest values were clustered in Africa and Asia. Many of the countries with the largest increases between the beginning and end of the record were European countries already with indicator values among the highest globally (e.g., Norway, Switzerland, Finland, Sweden).

The distribution of the biophysical indicator was left-skewed or bimodal throughout the record (Figure 3). At the beginning of the record, 41% of countries had biophysical capacity indicators less than the threshold (0.5) indicating limited capacity. This increased to 47% by the end of the record. The median indicator declined from 0.7 to 0.58 (Figure 4). The highest values of biophysical capacity were in Africa, Eastern Europe, South America, and the United States. Western and northern European countries have lower biophysical capacities because they lack spare arable land through which agriculture can be extensified (Fader *et al* 2016). Despite this patterning, the declines in biophysical capacity have been spread relatively evenly between continents.

Production diversity had a unimodal distribution throughout the record (Figure 3). The median diversity index for the beginning and end of the record, 46 and 47. Many of the biggest gains in the diversity index occurred in Africa and the Middle East. China, the United States, and several other countries with temperate or Mediterranean climates maintained high productivity diversity throughout the time-series. In contrast, many countries in Africa, and areas with semi-arid and the tropical climates had lower production diversity. The positive, but weak relationship between the socio-economic indicator and production diversity also suggests that wealthier nations are more likely to have higher production diversity but with large variations in this relationship.

Collectively, geographic patterns and lack of strong correlation between indices demonstrate that there are few countries with high values for all three dimensions of resilience considered in this analysis. Hence, our analysis shows different countries, and in many cases different regions, are resilient (or lack resilience) in different ways.

## 4 Discussion

The application of the resilience concept in the context of food security has become more frequent both in the academic and policy arenas (Pingali *et al* 2005, Suweis *et al* 2015, Béné *et al* 2016). Our analysis adds to these developments by evaluating factors of resilience contributing to country-scale food security of nations around the world, including country-specific diversity and redundancy of agricultural production and the food purchasing power of the poor. These indicators are available in time series based on standardized data, which allows for the evaluation of inter-annual variability and longer-term changes. Hence, our results contribute to filling a gap in the food security-resilience literature, which is dominated by local-scale studies based on individual hunger events (Béné *et al* 2016).

Our approach focuses on dimensions of resilience and not on estimating or reducing numbers of undernourished people. This difference in goals can cause interpretations that run counter to common. One example is that in our biophysical capacity index we consider having high yield gap as high resilience, whereas reduction of the yield gap is typically identified as a goal to feed the growing human population (Godfray *et al* 2010). While we agree with this interpretation of the yield gap issue, our approach notes there is a trade-off whereby yield gap reductions limit the transformative capacity in the sense that transformation of agricultural systems through intensification is no longer a viable option to increasing food production. Similar reasoning applies to extensification in terms of the amount of viable farmland currently in production where the production system become more rigid in the sense that it is operating on nearly all potentially arable land, reducing buffer area (Fader *et al* 2016).

Our social-economic indicator is similar to the “share of food expenditure by the poor” index of food security calculated by the FAO. However, the FAO indicator is only available for a small number of countries and years, which limits the potential to track geographic and temporal patterns. We were able to calculate our socio-economic indicator for 96 countries from 1992 to 2011 and these data are available on Github (doi: 10.5281/zenodo.192394). A limitation of our socio-economic indicators is that it compares the average per capita income of the lowest 20% of the population to the overall average food price index. It is probable that, for households, income and food expenditures are correlated (e.g. Kirkpatrick and Tarasuk 2003). A more comprehensive picture of food security among the poor would be gained by adding a measure of average food expenditures of the poor as a percentage of total income, which would provide a proxy for food access issues and tradeoffs with other essential expenditures (Misselhorn 2005). Such disaggregated data is not widely available, hence this indicator reflects variation in the ability to buffer price shocks by reducing non-food expenditures, but not the specific amount of money spent or food actually acquired (Timmer 2000). Overall, the socio-economic indicator relates to the absorptive coping capacity of the poor, especially in developing countries, and our study has expanded the potential to evaluate this aspect of resilience geographically and over time (Timmer 2000, Béné *et al* 2016).

The production diversity indicator in our analysis relates to the absorptive and adaptive capacities of agricultural production, which are key dimensions of resilience, while the biophysical capacity accounts for the ability of the system to transform agricultural systems through intensification or extensification. How these characteristics play out in practice depends on local factors. For example, Japan has little ability to transform its food production system because of lack of arable land for extensification. Many African countries, like Angola and Ghana have a high biophysical capacity but the actual ability to transform agricultural systems depends on the strength of local institutions, the ability to raise capital to convert land for agriculture and implement technologies and strategies for sustainable intensification like integrated crop water management, and the cultural acceptance of change (Béné *et al* 2016, Jägermeyr *et al* 2016, MacDonald *et al* 2016). On the other hand, a country like Japan may have strong institutions and large amounts of capital, but the biophysical limits of the country will always constrain the transformability of agricultural production. Connecting our indicators with the specific economics, governance, institutions, and cultures of every country is beyond the scope of a single paper. However, these examples demonstrate both the utility of the global context contributed by our analysis, as well as the need to integrate across scales and socio-environmental factors, to have a complete picture of resilience in the global food system.

Our analysis does not explicitly account for the influence of international trade. Twenty-four percent of food produced globally is traded between countries and the specific patterns of trade connections between countries may amplify or muffle the transmission of production shocks to consumers (D’Odorico *et al* 2014, d’Amour *et al* 2016, Marchand *et al* 2016). The actual impact of trade-related shocks reflects a variety of factors, but a key one is the self-sustainability of crop production for a variety of crops that are consumed domestically (d’Amour *et al* 2006). To a large extent, our production diversity index reflects the ability of a country to be self-sufficient and to be self-sufficient for a variety of commodities, and hence integrates some of the key factors influencing vulnerability to shocks propagated through trade. Other factors include the numbers of people living in extreme poverty and this is, to some extent, integrated within our socio-economic indicator (d’Amour *et al* 2006). Analyses of cereal trade networks and fish trade networks have identified certain regions, especially Central and West Africa, as susceptible to trade shocks (Gephart *et al* 2016, Marchand *et al* 2016). Our analysis finds that many of these countries have low socio-economic indicators values (where available), low production diversity, but high biophysical capacity. Hence our results reflect the influence of trade on resilience and emphasize the complex nature of food systems’ resilience.

## 5 Conclusions

Achieving food security requires food production and distribution systems that are resilient to disruption. This study provides national-scale indicators of food systems resilience with global coverage from 1992 to 2011. Our overall finding is that very few countries have exclusively high or low values for all dimensions, emphasizing the complexity and heterogeneity of the global food system. These indicators create the opportunity for global comparisons of resilience between nations, and provide context for developing generalizations about the resilience in the global food system.

## Figures and Tables

**Table 1.** Correlations between indicators were weak indicating that they are capturing redundant information. Kendall’s τ correlation coefficients are given in the upper right of the matrices and the corresponding probability values are given in the lower left.

Beginning of Record (1992–1996)			
	Socio-Economic	Biophysical Capacity	Production Diversity
Socio-Economic	---	τ = −0.01	τ = 0.23
Biophysical Capacity	p = 0.48	---	τ = 0.17
Production Diversity	p < 0.01	p =0.84	---
End of Record (2007–2011)			
	Socio-Economic	Biophysical Capacity	Production Diversity
Socio-Economic	---	τ = −0.06	τ = 0.18
Biophysical Capacity	p = 0.37	---	τ = −0.01
Production Diversity	p < 0.01	p = 0.92	---
